# Investigation of metabolic changes in STZ-induced diabetic rats with hyperpolarized [1-13C]acetate

**DOI:** 10.14814/phy2.12474

**Published:** 2015-08-13

**Authors:** Ulrich Koellisch, Christoffer Laustsen, Thomas S Nørlinger, Jakob Appel Østergaard, Allan Flyvbjerg, Concetta V Gringeri, Marion I Menzel, Rolf F Schulte, Axel Haase, Hans Stødkilde-Jørgensen

**Affiliations:** 1 Institute of Medical Engineering, Technische Universität MünchenMunich, Germany; 2 Department of Clinical Medicine, Aarhus UniversityAarhus, Denmark; 3 Department of Endocrinology and Internal medicine, Aarhus University HospitalAarhus, Denmark; 4 Danish Diabetes AcademyAarhus, Denmark; 5 Nuklearmedizinische Klinik und Poliklinik, Klinikum rechts der Isar, Technische Universität MünchenMunich, Germany; 6 GE Global ResearchMunich, Germany

**Keywords:** Acetate, diabetes mellitus, hyperpolarization

## Abstract

In the metabolism of acetate several enzymes are involved, which play an important role in free fatty acid oxidation. Fatty acid metabolism is altered in diabetes patients and therefore acetate might serve as a marker for pathological changes in the fuel selection of cells, as these changes occur in diabetes patients. Acetylcarnitine is a metabolic product of acetate, which enables its transport into the mitochondria for energy production. This study investigates whether the ratio of acetylcarnitine to acetate, measured by noninvasive hyperpolarized [1-^13^C]acetate magnetic resonance spectroscopy, could serve as a marker for myocardial, hepatic, and renal metabolic changes in rats with Streptozotocin (STZ)-induced diabetes in vivo. We demonstrate that the conversion of acetate to acetylcarnitine could be detected and quantified in all three organs of interest. More interestingly, we found that the hyperpolarized acetylcarnitine to acetate ratio was independent of blood glucose levels and prolonged hyperglycemia following diabetes induction in a type-1 diabetes model.

## Introduction

**Figure 1:**
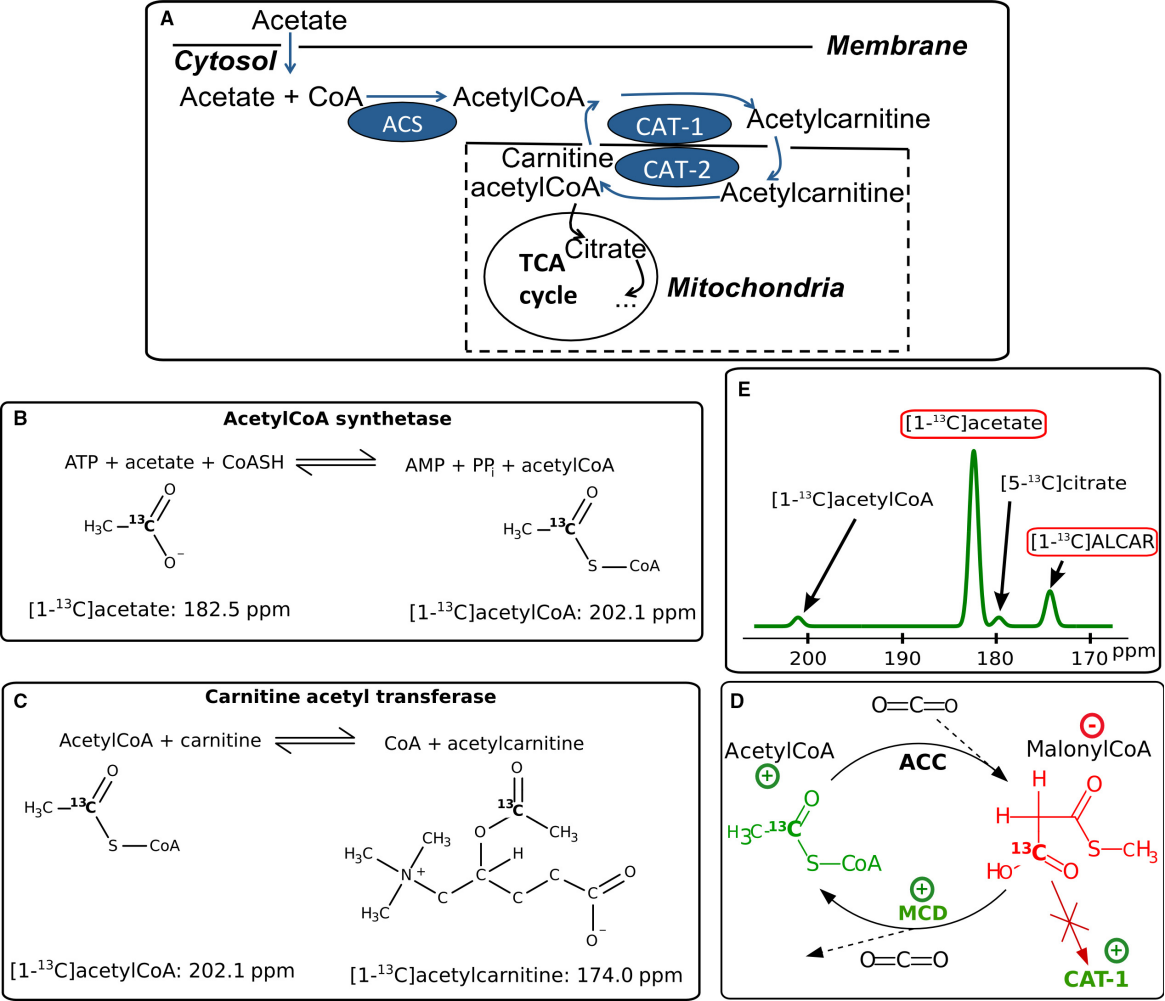
(A) Overview of the acetate metabolism: Acetate enters the cytosol where it gets converted to acetylCoA via acetyCoA synthetase (ACS). Then acetylCoA gets converted via carnitineacetyltransferase (CAT)-1 to acetylcarnitine, which can be shuttled into the mitochondria. There it can be metabolized in the TCA cycle, transformed back to acetylCoA via CAT-2. In diabetes myocardial CAT-1 activity is increased, which is a crucial step for myocardial fuel selection. (B) Acetylation of CoA via ACS. (C) CAT catalyses the formation of free CoA and ALCAR. (D) The enzyme activities of acetylCoA carboxylase (ACC) and malonylCoA decarboxylase (MCD) balance the activity of CAT-1. MCD is more active under diabetes, leading to more decarboxylation of malonylCoA, the lowered malonylCoA concentration leads to less inhibition of CAT-1. (E) Schematic spectrum showing the ^13^C chemical shifts of the molecules occurring in the metabolism of [1-^13^C]acetate.

Diabetes mellitus is one of the most common chronic diseases, with late complications affecting multiple organs. Diabetes comes with changes in cardiac fuel selection where reduced pyruvate activation via PDH has been observed (Kerbey et al. 1976), which has been demonstrated in studies using hyperpolarized ^13^C pyruvate (Schroeder et al. 2008) (Le Page et al. 2015). As a consequence of fuel selection changes, rates of fatty acid metabolism are increased (Lopaschuk 1996). Free Coenzyme A (CoA) and acetate are esterified in a glycolysis independent pathway. Thus, acetate as a marker for short-chain fatty acid metabolism may be capable of providing additional valuable information on enzymatic changes (Jensen et al. 2009). ^11^C acetate is a well-known tracer for Positron Emission Tomography (PET) in the heart (Grassi et al. 2012) (Klein et al. 2001), through which the uptake and clearance rate of acetate can be measured. More recently, hyperpolarized [1-^13^C]acetate metabolism has been quantified in skeletal (Bastiaansen et al. 2013) and cardiac muscle in rats (Jensen et al. 2009) (Koellisch et al. 2014). Furthermore, the turnover of hyperpolarized acetate to acetylcarnitine (ALCAR) has been quantified in healthy porcine hearts (Flori et al. 2014). Acetate has been used as a PET tracer for the kidneys, where it is metabolized but not excreted (Juillard et al. 2007). It has previously been reported that declining kidney function is correlated with reduced acetate uptake and renal clearance in patients with diabetic nephropathy (DN) (Shreve et al. 1995). Acetate can be metabolized by renal (Berg et al. 2011) and liver cells (Wolfe 2005) as well as by myocardial muscle (Bastiaansen et al. 2013) (Koellisch et al. 2014). Figure * *1A schematically depicts acetate metabolism. Acetate is transported into the cytosol, and converted to acetyl coenzyme A (AcCoA) by acetyl-CoA synthetase (ACS) (Fig.1B). The concentration of ACS is high in the myocardium and renal cortex, but lower in the liver (Knowles et al. 1974). AcCoA can be further converted to ALCAR, which is catalyzed by carnitine acetyl transferase1 (CAT-1) on the outer mitochondrial membrane (Berg et al. 2011) (Fig.1C). ALCAR can then be shuttled into the mitochondria, converted back into AcCoA (via CAT-2), and enter the TCA cycle. The cytosolic AcCoA pool is strongly regulated, and is held at levels more than ten times smaller than those of ALCAR (Schroeder et al. 2012). This leads to a very low signal to noise ratio (SNR) of this intermediate metabolite in magnetic resonance spectroscopy, even when hyperpolarized compounds are used. The chemical shift difference between [5-^13^C]citrate and [1-^13^C]acetate is only ≈90 Hz at 3T. This impairs the in vivo separation of the citrate signal from the acetate signal at 3T due to the large concentration difference of the two molecules. Therefore, the focus of this study was set to quantify [1-^13^C]acetate and [1-^13^C]ALCAR signals (Fig.1E).

ACS is present in two forms, cytosolic enzyme ACS-1 and mitochondrial enzyme ACS-2, which have similar affinities to acetate, but are independently regulated (Fujino et al. 2001). In diabetes, ACS-1 activity is reduced in the liver, which switches to acetate excretion rather than consumption, and thus increases the blood concentration of acetate (Wolfe 2005). Cardiac ACS-1 activity has been reported to be unchanged in diabetic rats, whereas ACS-2 is induced under diabetic conditions in heart and skeletal muscles (Fujino et al. 2001). The method described herein is able to quantify the cytosolic conversion of acetate toward ALCAR, influenced by cytosolic ACS-1.

In diabetes mellitus, the up-regulation of fatty acid consumption at the cost of glycolysis is mediated by CAT-1 (Lopaschuk et al. 2010). This key enzyme is effectively inhibited by malonylCoA, whose concentration is dependent on blood insulin levels (Awan and Saggerson 1993). In diabetes, more malonylCoA is decarboxylated leading to a decreased concentration of malonylCoA in diabetic myocardium and thus less inhibition of CAT-1 (see Fig.1D) (Lopaschuk et al. 2010). This has led us to hypothesize that myocardial conversion of acetate to ALCAR might be increased in diabetes. As mentioned above, the consumption of acetate was reported to be reduced in diabetic kidneys (Shreve et al. 1995). However, it is not clear whether there is a decrease in the activation of acetate via AcCoA conversion to the membrane transportable molecule ALCAR.

Acetate, which is the fatty acid with the shortest carboxylic chain, as a marker for fatty acid metabolism cannot be used to investigate the *β*-oxidation of fatty acids. Fatty acids with longer chains such as [1-^13^C]butyrate (Ball et al. 2014) and [1-^13^C]octanoate (Yoshihara et al. 2015) have been used in dynamic nuclear polarization (DNP) experiments, and these substrates were used to reveal information about cardiac metabolism in perfused rat hearts and in vivo. However, hyperpolarization (HP) studies with longer fatty acids suffer from lower polarization levels and shorter relaxation times as compared to acetate.

In this work, we aimed to determine whether the ratio of acetylcarnitine to acetate measured after injection of hyperpolarized [1-^13^C]acetate could serve as an in vivo marker for myocardial, hepatic, and renal metabolic changes in streptozotocin (STZ)-induced diabetes in rats using a setup similar to human hyperpolarized MR setup.

## Materials and Methods

### Animals

A group of eight-week-old female Wistar rats (*n* = 12) (Taconic, Ry, Denmark) weighing 178.5 ± 8.4 g were included in this study. Rats were randomized assigned to the control group (*n* = 5) or diabetes group (*n* = 7). Diabetes was induced by an intravenous injection of STZ (55 mg/kg body weight; Sigma-Aldrich, St. Louis) dissolved in 10 mM cold citrate buffer (pH 4.5) as previously described (Laustsen et al. 2013) (Laustsen et al. 2014). The MR scans were performed 3 weeks after injection. Blood glucose of each animal was measured in tail-capillary blood with a Contour blood glucose meter (Bayer Diabetes Care, Copenhagen, Denmark). All animals had unrestricted access to water and standard chow throughout the study. The rats were kept in cages with a 12:12-h light dark cycle, a temperature of 21 ± 2°C, and a humidity of 55 ± 5%. Before the MR-session, the diabetic rats had an average blood glucose concentration of 15.9 ± 5.9 mmol/L and a body weight of 216 ± 9 g. The healthy control group had blood glucose concentration of 7.5 ± 0.3 mmol/L and weighed 222 ± 13 g.

During the MR-session, the animals were anesthetized via continuous inhalation of 2.5% of sevoflurane in oxygen. The rats were kept warm with an air heating system, and the body temperature ahead of the injection was 37.20 ± 0.37°C with a respiration rate of 55.1 ± 10.8/min. Blood oxygenation was monitored with a blood oximeter, and due to the continuous inhalation of gas the blood oxygenation of all rats was close to saturation (98–100%). These physiological parameters did not differ between the healthy and the diabetic groups. This study was performed in compliance with the guidelines for use and care of laboratory animals and was approved by the Danish Inspectorate of Animal Experiments.

### 13C MR acquisition

**Figure 2:**
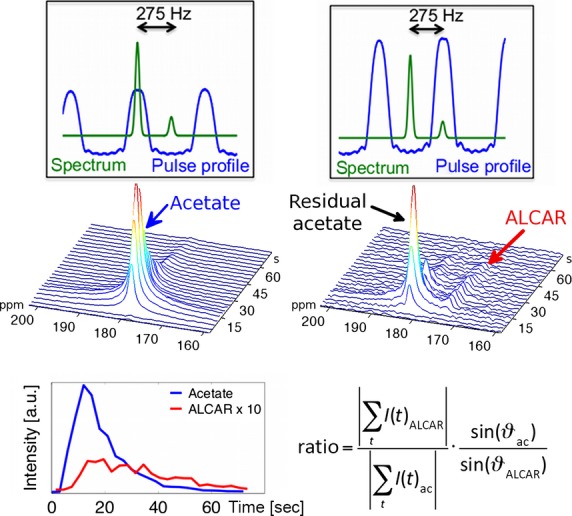
First row: With an alternately excitation of acetate with 4° and ALCAR with 15° two sets of spectra are acquired (blue: spectral pulse profile; green: schematic spectrum). Second row: Representative spectra after acetate and ALCAR excitation. The ALCAR spectrum is dominated by the residual acetate signal at the first timepoints. However, the two signals are well separable. Third row: Time-course of the signal intensities (left) and quantification of the signal ratio with flip angle correction (right).

A spectro-spatial (SPSP) pulse was used in order to excite acetate and ALCAR separately and with different flip angles. This allows for a good separation of the peaks at the time during or shortly after injection. The acetate concentration is up to twofold higher than the concentration of ALCAR. Therefore, a nonselective pulse in the spectral domain would lead to an overlap of the ALCAR and acetate signals. As the polarization of the substrate depletes with every excitation with the flip angle *ϑ* by a factor of cos(*ϑ*), it is favorable to acquire the substrate’s signal with a lower flip angle than the metabolite signal (Fig.* *2). This approach saves magnetization during acquisitions of the substrate, where the SNR is much higher. An SPSP-pulse, designed according to (Schulte and Wiesinger 2013) for a previous HP [1-^13^C]acetate study was used and is described in (Koellisch et al. 2014) in more detail.

Slice selective spectra of three slices of 12-mm thickness containing the heart, liver, and kidneys, respectively, were acquired in each animal with a *T*_*R*_ of 3 sec for each metabolite. The flip angle was set to 4° for the acetate and 15° for the ALCAR excitation.

Additionally, in four animals (two healthy, two diabetic) time-resolved acetate maps were acquired with a second acetate injection approx. 20 min after the first injection in order to investigate the acetate distribution in the organs of interest. Therefore, a single arm spiral trajectory over a 8 × 8 cm^2^ FOV and a real resolution of 5 × 5 mm^2^ was applied with the flip angle *ϑ* = 10°, slice thickness of 12 mm, and repetition time *T*_*R*_* *= 3 sec triggered on respiration. The trajectory and reconstruction have been previously described (Koellisch et al. 2014). As a reference for these images, ^1^H gradient echoes for each slice were recorded ahead of the injection. Automatic shimming was performed during the prescan of the proton scan, and for the ^13^C resonance, a linewidth (FWHM) below 1 ppm (32 Hz) was reached in each slice of interest.

All MR experiments were performed on a 3T GE HDx scanner (GE Healthcare, Milwaukee, WI). The proton reference scans and the ^13^C scans were acquired with a dual-tuned ^1^H-^13^C-volume coil for radiofrequency transmission and reception (Derby et al. 1990).

### Quantification

The spectra of acetate and ALCAR during the first 60 sec after the start of the acetate injection were flip angle corrected and summed. The total signal ratio of ALCAR/acetate was calculated from the absolute value of this sum (see Fig.2). Additionally, an in vivo estimation of the relaxation time was extracted from the data. Therefore, the acetate intensities from the spectroscopy study starting 20 sec after injections were evaluated and fitted to a monoexponential decay curve. The last intensity considered for this fit was determined by the first SNR value below 3. The fitting result was considered appropriate if the fitted data included at least eight signal intensities. As this fit does not distinguish relaxation from metabolism, the result of the fit is called the apparent longitudinal relaxation time (*T*_1,app_). Finally, *T*_1,app_ was accounted for the signal loss due to acetate excitation according to (Schilling et al. 2013), which only has a minor effect due to the low flip angle.

### Statistics

The values of the signal ratios were analyzed for normal distribution by a quantile–quantile plot. Values differing from the normal distribution by a *P*-value of 0.05 were excluded and marked as outliers. For a statistical investigation of the differences in the ratios of the healthy and diabetic groups, an unpaired Student’s *t*-test was applied.

### Substrate preparation

The substrate was prepared by mixing 48 wt% of sodium [1-^13^C]acetate with 30 wt% water 22 wt% of glycerol, and adding 15 mM Oxo63 radical (Bowen and Ardenkjaer-Larsen 2013). 135 mL of the 7.7 M acetate mixture were polarized in a 5T SPINLab (GE Healthcare, Brøndby, DK) for 180 min. With this procedure, a liquid polarization level of approx. 20% was reached, and the acetate relaxation time in the dissolution medium measured during polarization tests was 65 sec at 3 T. The probe was dissolved rapidly in 7.5 mL of aqueous phosphate-buffered saline (1×), which provides a solution with a physiological temperature and pH. 10 sec later, 1 mL of the hyperpolarized 137 mM acetate solution was injected via a catheter into the rats’ tail vein with an injection speed of approx. 125 *μ*L/sec. Taking the average weight of the rats into account, the acetate concentration in the animals blood can be estimated to be around 10 mM (Lee and Blaufox 1985).

## Results

**Figure 3:**
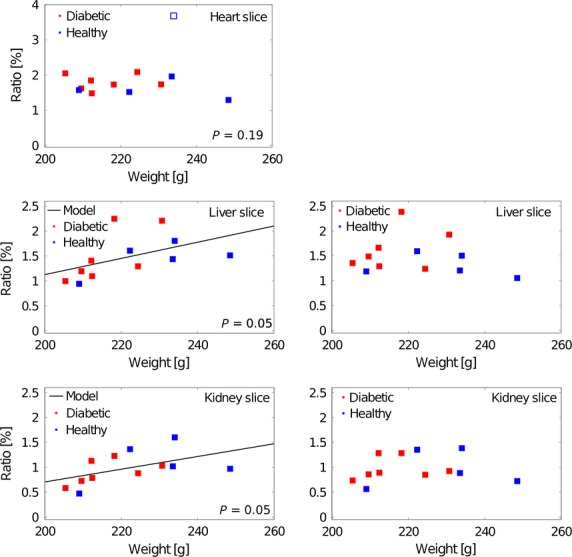
The ALCAR/acetate ratio is significantly correlated with the rats’ weight for the liver and the kidney slices, but not for the heart slice (left column). Accounting for this effect, the results for liver and kidney slice were corrected by interpolating them to the average weight (right column).

The measured ALCAR/acetate ratios in the heart, the liver and the kidney slice are illustrated against the weight of the rats in Figure * *3. In the heart slice one data point was excluded as an outlier and was not considered for the statistical analysis. A significant positive linear correlation between the weight and the ratio was observed for the liver (*r* = 0.49, *P* = 0.05) and the kidney (*r* = 0.51, *P* = 0.05) slice. The results were corrected for this linear effect by interpolating them to the average weight. In the heart slice there was no significant correlation perceptible.

**Figure 4:**
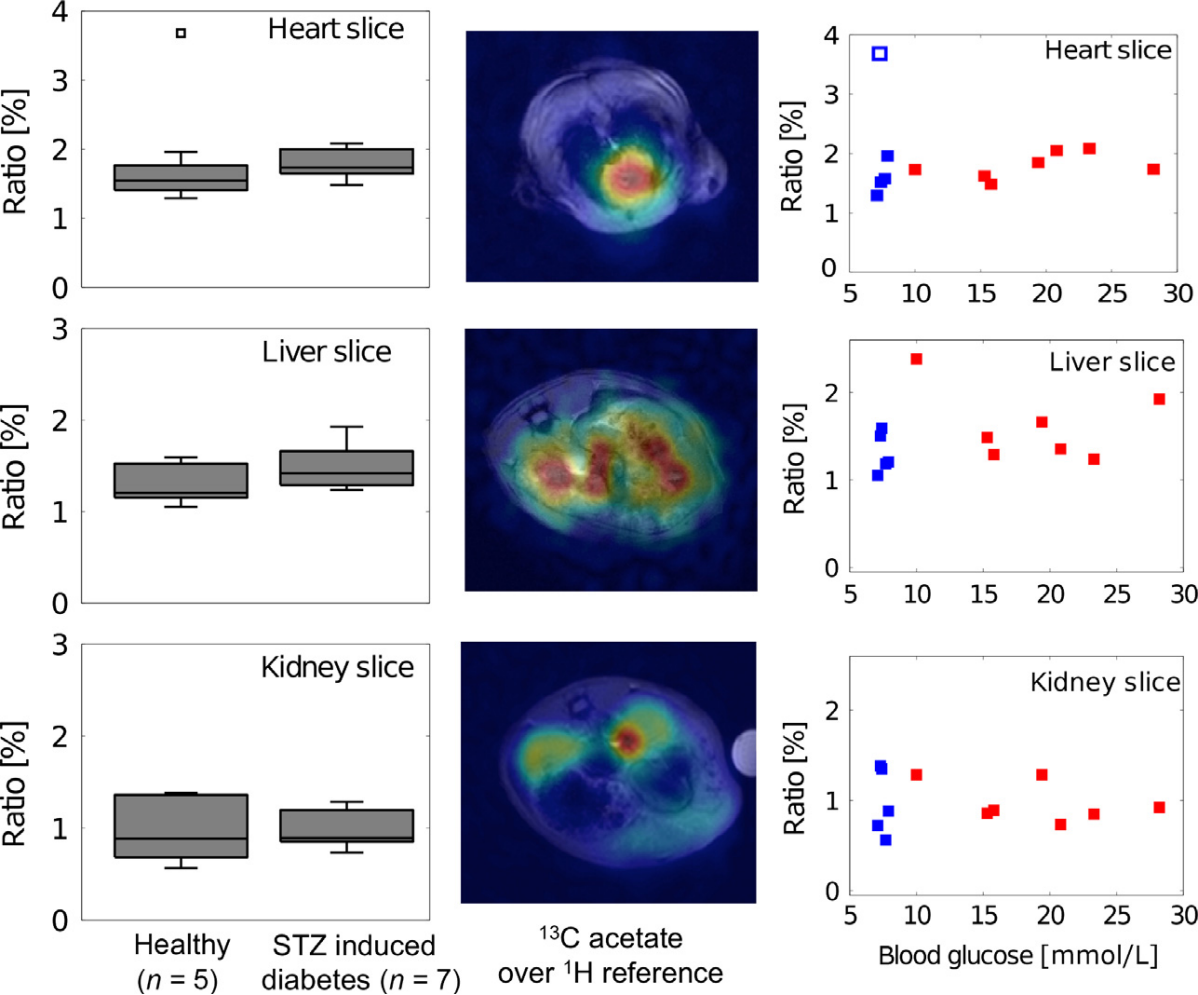
ALCAR/acetate ratio in healthy and diabetic rats. The 1st column shows boxplots containing median, 25 and 75 percentiles as well as the maximal and minimal value for each slice. The 2nd column illustrates an example of the acetate distribution in the three slices. In the 3rd column the ratio is plotted versus the blood glucose concentration for the healthy (blue) and the diabetic (red) animals.

With these data in all three slices no statistical significant change between the healthy and the diabetic group is perceptible (Fig.* *4, first column). The *P*-values calculated with the unpaired Student’s *t*-test (stated in Table* *1) confirm that there is no statistically significant difference between the two groups. Furthermore, the mean and the standard deviation of the values measured in the healthy (*n* = 5) and the diabetic (*n* = 7) rats are stated in Table* *1.

**Table 1:** ALCAR/acetate ratio in the healthy and diabetic groups for the three slices investigated. The *P*-values were calculated by an unpaired Student’s *t*-test. The apparent longitudinal relaxation time *T*_1,app_ was averaged over all rats

Slice	Ratio healthy (*n*)	Ratio diabetic (*n*)	*P*-value	*T*_1,app_ (*n* = 12)
Heart	1.59 ± 0.28% (4)	1.79 ± 0.22% (7)	0.11	12.4 ± 1.2 sec
Liver	1.41 ± 0.23% (5)	1.62 ± 0.41% (7)	0.16	14.3 ± 2.6 sec
Kidney	0.98 ± 0.37% (5)	0.98 ± 0.22% (7)	0.50	14.0 ± 1.7 sec

In the second column of Figure* *4*,* exemplary acetate maps for the three slices are shown with the proton reference images as backgrounds. These maps show the averages over three excitations acquired 15 to 24 sec after injection in a diabetic animal. The images demonstrate that the substrate is well distributed in each organ of interest, whereas the signal from skeletal muscle regions is negligible.

Furthermore, the ALCAR/acetate signal ratios were compared to the measured blood glucose levels (third column Fig. * *4). In all of the three organs of interest, no significant correlation was observed.

The apparent longitudinal relaxation times *T*_1,app_ fitted for each slice and averaged over all (*n* = 12) animals are shown in Table* *1. In all three slices, the relaxation times did not differ between the healthy and diabetic groups. In the heart slice, *T*_1,app_ is slightly shorter (12.4 ± 1.2 sec) than in the other organs (liver 14.3 ± 2.6 sec, kidneys 14.0 ± 1.7 sec). The in vivo *T*_1,app_ averaged over all three slices, neglecting conversion through metabolism of [1-^13^C]acetate at 3 T, was 13.7 ± 2.1 sec.

## Discussion

In this study, we demonstrate that it is feasible to quantify the acetate to ALCAR metabolism in a clinical setup using a human 3T MR-system in combination with a polarizer authorized for human studies. The primary limitation of this study was the relatively large variation of the ALCAR/acetate ratio, which was due to the low SNR of the ALCAR-data. In general, the conversion rates reported for acetate (Bastiaansen et al. 2012, 2013; Koellisch et al. 2014) are much lower than the rapid conversion rates of pyruvate to lactate or alanine, or its rapid oxidation to CO_2_. This leads to a limited amount of ALCAR signal accumulation in the relatively short time window of an HP experiment, and thus hinders the potential of clinical application.

The correlation of the ALCAR/acetate ratio with the rats’ weight in the liver and the kidney slice is prospectively due to saturation effects of ACS with increasing substrate concentration. For the cardiac slice this behavior was not observed, here the ALCAR production scales with the substrate concentration. This supposes, that the cytosolic concentration of acetate is small against the cardiac Michaelis Menten constant of ACS-1. For a quantitative analysis of the enzymatic kinetic in each organ, a series of experiments with a wide range of concentration value could be conducted, as it is proposed for skeletal muscle in (Bastiaansen et al. 2013).

The ALCAR to acetate ratio was not significantly affected 3 weeks after diabetes induction. This finding contradicts the expectation that cardiac oxygen consumption is directly linked to acetate turnover as seen with PET (Grassi et al. 2012) (Croteau et al. 2012). This strengthens the argument that the first step – the reaction from acetate to ALCAR via ACS – is the rate-limiting step. The expected increase in CAT-1 does not sufficiently affect the amount of ALCAR converted in the time range of interest (one minute) to detect this change given the high variance of the data. The accuracy of the measurement could potentially be increased by combining higher field strength with surface coils targeting each organ of interest. Proton decoupling during the ^13^C acquisition could prevent peak splitting, and thus lead to sharper lines with better SNR, albeit this was technically not feasible in this setup.

As ALCAR also is an intermediate product of acetate metabolism, it could be that more ALCAR gets produced due to higher CAT-1 activity, while at the same time the conversion away from ALCAR toward the TCA-cycle inside the mitochondria is increased. This would reduce the ALCAR pool and therefore decrease the measured signal ratio. With the current setup, this cannot be further investigated due to the small chemical shift between [1-^13^C]acetate and [5-^13^C]citrate. The separation of these signals could be improved using an MR-scanner with a higher B_0_ field strength. The use of short fatty acids with longer carboxylic chain, like [1-^13^C]butyrate or [1-^13^C]octanoate, which have higher chemical shift differences compared to the TCA cycle products citrate and glutamate (Ball et al. 2014) (Yoshihara et al. 2015), could further enable the detection of TCA cycle activity. Due to the lower polarization and the shorter *T*_*1*_-time compared to [1-^13^C]acetate, this approach has potential in a preclinical setup with higher sensitivity.

In the kidney slice no statistically significant change was observed. The same held true for the liver, where the variation of the data was larger since the SNR of ALCAR was lower than that in the heart and kidney slices. Since no significant alteration of the ALCAR/acetate ratio was observed, this characteristic does not qualify as a potential clinical marker for diabetic changes.

In addition, this work reports on the in vivo measured apparent relaxation times of [1-^13^C]acetate. Neglecting metabolic conversion leads to an underestimation of the real *T*_*1*_-time. However, if one takes into account a maximal conversion rate of 6·10^-3^·sec^−1^ as reported in studies of the heart (Bastiaansen et al. 2012) and skeletal muscle (Bastiaansen et al. 2013), this systematic error is below 10%. The shorter *T*_*1*_ time in the heart could be due a greater conversion of acetate, but may also be due to a net flux out of the slice, especially out of the blood pool. In general, the relaxation rate measured in the heart is dominated by the blood because of the large pool of blood within the ventricles. In contrast to that in the kidney, the substrate accumulates during the experiments, and hence averaging over all three slices leads to a better estimation of the in vivo relaxation time. The *T*_1*,*app_ measured in this work was approximately 20% lower than that previously published for Na[1-^13^C]acetate at 3T measured in vivo in the porcine heart (Flori et al. 2014). Whether this was due to different formulations, different blood concentrations, or other physiological parameters remains unclear.

## Conclusion

Taken together, we demonstrate that the HP ALCAR/acetate ratio does not significantly change with blood glucose levels and prolonged hyperglycemia (3 weeks) following diabetes induction. Therefore, the current parameter described herein is not suitable as a marker for cardiac, renal, or hepatic metabolic changes occurring in early diabetes, with an experimental setup similar to human hyperpolarized MR setups. Future investigations at higher field strengths may yield more information about acetate metabolism, particularly about its contribution to energy production via the TCA-cycle.
